# Biochemical and Imaging Markers associated with the A431E *PSEN1* and V717I *APP* Mutations: A Comparison Study

**DOI:** 10.1002/alz70856_099134

**Published:** 2025-12-25

**Authors:** John M Ringman, James Luo, Yonggand Shi, Nasim Sheikh‐Bahaei, Helena C Chui, Dominantly Inherited Alzheimer Network (DIAN)

**Affiliations:** ^1^ Department of Neurology, Keck School of Medicine at USC, Los Angeles, CA, USA; ^2^ Keck School of Medicine, University of Southern California, Los Angeles, CA, USA; ^3^ Department of Neurology, Stevens Neuroimaging and Informatics Institute, Keck School of Medicine, University of Southern California, Los Angeles, CA, USA; ^4^ Department of Neurology, Keck School of Medicine, University of Southern California, Los Angeles, CA, USA; ^5^ Washington University in St. Louis, St. Louis, MO, USA

## Abstract

**Background:**

There are quantitative and some qualitative neuropathological differences between autosomal dominant AD (ADAD) and late‐onset AD, as well as differences among ADAD mutations. Using data from the DIAN observational study, we compared fluid, MRI, PiB and FDG PET biomarkers in carriers of the A431E *PSEN1* and V717I *APP* mutations at comparable clinical disease stages.

**Method:**

Using data from Data Freeze 15, participants were categorized as asymptomatic (CDR = 0), mildly symptomatic (CDR = 0.5) or having dementia (CDR > 0.5). We compared biomarkers between 17 carriers of the A431E PSEN1 mutation (CDR 0 = 7, CDR 0.5 = 7, CDR > 0.5 = 3) to 31 carriers of the V717I APP mutation (CDR 0 = 15, CDR 0.5 = 13, CDR > 0.5 = 3) using linear models adjusting for estimated years to symptom onset. SUVRs for PiB signal were standardized to the corpus callosum. For PiB SUVRs, p values < 0.01 were considered significant.

**Result:**

Among mildly symptomatic carriers, carriers of the A431E PSEN1 mutation had elevated plasma Abeta42 levels relative to carriers of the V717I APP mutation (49.4 vs. 35.1 pg/mL). Asymptomatic carriers of the V717I mutation had decreased cerebral metabolism relative to A431E mutation carriers, particularly in the nucleus accumbens, caudate, amygdala, and brainstem. Metabolic differences between mutation types were not detected in symptomatic cases. Among asymptomatic carriers, PiB SUVRs were greatest in A431E carriers relative to V717I carriers in pericalcarine cortex, parietal lobe, and thalamus. Among mildly symptomatic carriers, PiB SUVRs were elevated in the precuneus in carriers of the A431E mutation relative to the V717I mutation. Among demented carriers, persons with the V717I mutation had higher PiB SUVR values than A431E carriers in multiple brain areas, with the greatest differences being in the nucleus accumbens, caudate, and widespread areas of the frontal and parietal cortices. No robust differences were found in CSF or MRI volumetric measures between the two mutation types at any disease stage.

**Conclusion:**

There are differences in amyloid PET imaging measures between the A431E PSEN1 and V717I APP mutation which appear to be dependent on disease stage.

**Funding Sources**: U19AG032438, P30AG066530, R01AG06901, R01AG062007, U01AG051218

